# Field Evidence of *Fasciola hepatica*-Mediated Modulation of Antibody Responses to Foot-and-Mouth Disease Vaccination in Buffaloes

**DOI:** 10.3390/vaccines14010036

**Published:** 2025-12-28

**Authors:** Juan Manuel Sala, Maximiliano Wilda, María Cruz Miraglia, Mariángeles Castillo, Daniel Mariano Pérez-Filgueira, Teresa Freire, Alejandra Victoria Capozzo

**Affiliations:** 1 Instituto Nacional de Tecnología Agropecuaria, Estación Experimental Agropecuaria Mercedes, Juan Pujol al Este s/n, Mercedes, Corrientes 3470, Argentina; sala.juan@inta.gob.ar; 2 Consejo de Investigaciones Científicas y Tecnológicas (CONICET), Godoy Cruz 2290, Buenos Aires 1270, Argentina; maximiliano.wilda.cevhan@uai.edu.ar (M.W.); miraglia.maria@inta.gob.ar (M.C.M.); castillo.mariangeles@inta.gob.ar (M.C.);; 3 Centro de Virología Humana y Animal, CONICET—Universidad Abierta Interamericana, Buenos Aires 1270, Argentina; 4 Instituto Nacional de Tecnología Agropecuaria, Instituto de Virología e Innovaciones Tecnológicas (IVIT, INTA-CONICET), N. Repetto at De los Reseros S/N, Hurlingham 1686, Buenos Aires 1270, Argentina; 5 Laboratorio de Inmunomodulación y Vacunas, Unidad Académica Inmunobiología, Facultad de Medicina, Universidad de la República (UdelaR), Avenida General Flores 2125, Montevideo 11800, Uruguay; tfreire@fmed.edu.uy; 6 Centro de Altos Estudios en Ciencias Humanas y de la Salud, Universidad Abierta Interamericana, Montes de Oca 745, Buenos Aires 1270, Argentina

**Keywords:** helminth infection, immunomodulation, FMD vaccine, post-vaccination monitoring

## Abstract

Background: *Fasciola hepatica (F. hepatica)* infection reduces antibody avidity to foot-and-mouth disease virus (FMDV) vaccination in cattle despite preserved total antibody levels. However, its effect on vaccine-induced immunity in water buffaloes (*Bubalus bubalis*), which contribute to FMDV maintenance in endemic settings, has not been investigated. Objectives: To evaluate the effect of natural *F. hepatica* infection on the magnitude and functional quality of the FMDV–specific antibody response in buffaloes under field conditions. Methods: Two buffalo herds (*n* = 50 each) were classified by infection status using coproparasitological analysis and serology. All animals were vaccinated within the national foot-and-mouth disease control programme, with the last dose administered 264 days before sampling. Serum neutralising titres, total antibodies by liquid-phase blocking ELISA, IgG levels, and IgG avidity to the A24/Cruzeiro vaccine strain were determined. Results: *F. hepatica*-infected buffaloes exhibited consistent decreases across all vaccine-induced antibody parameters. Neutralising titres were reduced approximately two-fold, IgG avidity by about 38 percent, IgG levels by about 36 percent, and liquid-phase blocking ELISA titres by about 1.6-fold compared with non-infected animals. Conclusions: This study provides the first field evidence that fasciolosis is associated with changes in the magnitude and quality of vaccine-induced humoral responses following FMDV vaccination in water buffaloes, indicating that *F. hepatica* infection may influence the interpretation of post-vaccination serological monitoring in this species under endemic field conditions.

## 1. Introduction

In recent years, South America has made substantial progress toward the eradication of foot-and-mouth disease (FMD). Countries such as Brazil and Bolivia have already ceased vaccination, while others (including Argentina) continue preventive immunisation programmes. The decision to suspend vaccination remains closely linked to each country’s epidemiological situation and national policy framework. These developments reflect a changing regional landscape, particularly for countries like Argentina that maintain an FMD-free status with vaccination while neighbouring others that do not vaccinate.

Post-vaccination monitoring of foot-and-mouth disease virus (FMDV) immunity in water buffaloes (*Bubalus bubalis*) remains challenging, despite their inclusion in vaccination programmes in several endemic countries. Buffaloes can sustain subclinical FMDV infection and shed virus for prolonged periods, even following vaccination, which complicates disease surveillance and control efforts [1,2,3,4]. However, information on the magnitude and functional quality of vaccine-induced humoral responses in this species remains limited [5,6], and most serological assays currently applied have been validated for cattle rather than buffaloes.

In Argentina, water buffaloes are vaccinated with inactivated oil-adjuvanted FMDV vaccines as part of the national control programme and are primarily raised in wetland ecosystems, particularly in regions bordering Paraguay, Brazil, and Bolivia. These ecological conditions favour the persistence of endemic parasitic infections, including fasciolosis, which may influence immune responsiveness to vaccination. *F. hepatica* infection induces a regulatory immune environment characterised by Th2- and Treg-biased responses, which can interfere with the development of effective vaccine-induced immunity [7,8,9].

*F. hepatica* can alter the immune responses in cattle [10,11]. We recently demonstrated that experimental *F. hepatica* infection alters the humoral response to FMDV vaccination in cattle [6]. In contrast, limited information is available on the immunological consequences of fasciolosis in water buffaloes. Available data are limited to experimental studies with *F. gigantica*, which have reported modulation of Th1 responses, early suppression of antigen-presentation pathways, and altered cytokine profiles in buffaloes [12,13,14]. To date, no studies have assessed whether natural fasciolosis affects the magnitude or functional quality of humoral immunity induced by viral vaccination in field conditions.

The objective of this study was to determine whether natural *F. hepatica* infection modulates the humoral immune response to FMDV vaccination in water buffaloes under field conditions. This was addressed by comparing vaccine-induced antibody responses between parasite-free and *F. hepatica*-infected herds. The results indicate that parasitic infection may influence FMD vaccine responses in water buffaloes, a species of epidemiological relevance for FMD control in endemic regions.

## 2. Materials and Methods

### 2.1. Vaccines

Commercial tetravalent inactivated foot-and-mouth disease virus (FMDV) vaccines formulated as single oil emulsions were used in this study. Vaccines contained strains A24/Cruzeiro, A/Arg/2001, O1/Campos, and C3/Indaial and were produced by the same local manufacturer and were officially approved by the national regulatory authority (SENASA). In accordance with national regulations [15], vaccine approval certifies the absence of non-structural proteins and compliance with minimum potency requirements, defined as an Expected Percentage of Protection (EPP) ≥75% for homologous challenge in cattle. For strain A24/Cruzeiro, EPP values are based on established correlations between liquid-phase blocking ELISA and virus neutralisation test titres measured post-vaccination and protection following homologous challenge [16]. These titres are 1.90 for LPBE and 1.40 for VNT [16].

All animals were injected three times with a 2 mL vaccine dose, applied subcutaneously in the neck according to the manufacturer’s recommendations and official regulations from the National Agrifood Health and Quality Service (SENASA), Argentina [15].

### 2.2. Cells and Virus

Inactivated and concentrated preparations of A24/Cruzeiro FMDV strains were prepared as described before [6]. Baby hamster kidney (BHK) cells, strain 21, clone 13 (ATCC, INTA’s repository), were maintained as described previously and used to grow the vaccine virus strains provided by SENASA [15].

### 2.3. Animals and Experimental Design

Water buffaloes (*Bubalus bubalis*) from two farms located in Corrientes Province, Argentina, were included in the study. Farm A (*n* = 50) was free of *Lymnaea* snails, and animals were negative for *F. hepatica*, whereas Farm B (*n* = 50) comprised naturally infected animals. Animals had received routine vaccinations in previous campaigns, and individual serum samples were collected 264 days after the last (third) vaccination.

Buffaloes from both farms tested negative for bovine viral diarrhoea virus antigen and antibodies using commercial ELISA kits and were also negative for *Neospora caninum* by in-house ELISA. Animals were free from brucellosis and tuberculosis according to routine herd health records. Individuals were selected using random number assignment.

Blood sampling was performed in accordance with animal welfare regulations under protocol no. 25/2013, approved by the Institutional Committee for the Use and Care of Experimental Animals (CICUAE), CICVyA-INTA.

### 2.4. Identification of F. hepatica Infection

Farms were screened for the presence of the intermediate snail host of fasciolosis (*Lymnaea* spp.). Based on these surveys, two farms were selected: one without detectable snails (Farm A) and one with confirmed presence of *Lymnaea* spp. (Farm B). Faecal samples were collected from randomly selected buffaloes from each farm using random number assignment. The presence of *F. hepatica* infection was assessed by faecal egg detection using the sedimentation technique [17,18] and confirmed by serological analysis. Anti-*F. hepatica* IgG levels were measured using an in-house ELISA based on plates coated with *F. hepatica* total extract, as previously described [19]. Antibody avidity was evaluated using the same ELISA with an additional urea-elution step. After serum incubation, plates were treated for 20 min with PBS, 5 M urea, or 7 M urea to dissociate low-avidity antibodies. The avidity index (AI) was calculated for samples with detectable antibody levels as the ratio of the signal obtained after 7 M urea treatment to that obtained after 5 M urea treatment, multiplied by 100. Serum dilutions (1:500 and 1:1000) were selected to ensure measurements were within the linear range of the assay [19].

### 2.5. Serology Against FMDV

#### 2.5.1. Detection of Antibodies Against Non-Structural Proteins (NSPs)

Antibodies to FMDV non-structural proteins were detected using a commercial ELISA kit (K-3ABC, CEVHAN, Buenos Aires, Argentina) following the manufacturer’s instructions [20].

#### 2.5.2. Liquid-Phase Blocking ELISA

Total antibodies against FMDV serotype A (A24/Cruzeiro) were quantified by liquid-phase blocking ELISA in accordance with the WOAH Manual, using a commercial kit (K-FLP-A24, CEVHAN) [21].

#### 2.5.3. Neutralisation Assay

FMDV-neutralising antibodies in serum of vaccinated buffaloes were titrated by a conventional virus microneutralisation test (VNT) using an infective culture adapted A/24 Cruzeiro strain (10^7^ TCID50/mL) on BHK-21 as described before [6]. Neutralising antibody titres were expressed as the log10 serum dilution neutralising 50% of the virus inoculum, according to the Reed and Müench method.

#### 2.5.4. Single Dilution Indirect and Avidity ELISAs

Assessment of FMDV-specific total IgG and IgG avidity using single dilution indirect ELISA was performed as described by Lavoria et al. [22] and adapted to buffalo samples according to Sala et al. [6]. Briefly, samples were run by duplicates diluted 1:50 (final dilution): one of the wells was washed with PBS and the other with PBS 6 M urea to detach low-avidity binders. OD values for samples and controls were corrected by subtracting mean blank OD values (cOD and a ratio to untreated samples were estimated).

### 2.6. Statistical Analysis

The D’Agostino–Pearson omnibus normality test was used to test how much our dataset deviates from a normal distribution by assessing both its skewness (asymmetry) and kurtosis (tailedness). Differences between infected and control animals were estimated using the Mann–Whitney test. Antibody titres induced after vaccination and measured by LPBE and VNT were also referred to the 75% Expected Percentage of Protection (EPP) already established for cattle for the A24/Cruzeiro strain as Log_10_ titres 1.9 and 1.4, respectively. Throughout the analysis, asterisks indicate statistically significant differences: * *p* < 0.05; ** *p* < 0.01; *** *p* < 0.001.

Analysis was performed using GraphPad Prism 10.2.2 (Prism, La Jolla, CA, USA).

## 3. Results

### 3.1. F. hepatica IgG Levels

Serum samples were collected 264 days after the third immunisation from two buffalo farms, either infected or not infected with *F. hepatica*.

The farm classified as “free from fasciolosis” (Farm A) showed no detectable *F. hepatica* eggs in any of the faecal samples, and all animals exhibited undetectable levels of anti-*F. hepatica* IgG. The intermediate host, *Lymnaea* spp., was not detected in the farm area. In contrast, all animals from the farm where snails were present (Farm B) showed positive detection of *F. hepatica* eggs in faecal samples. Only 7 of the 50 animals had no more than one detectable egg in their faeces on the day of sampling, and only two of these animals exhibited low anti-*F. hepatica* IgG levels, confirming that this was a naturally *F. hepatica*-infected herd.

**Figure 1:**
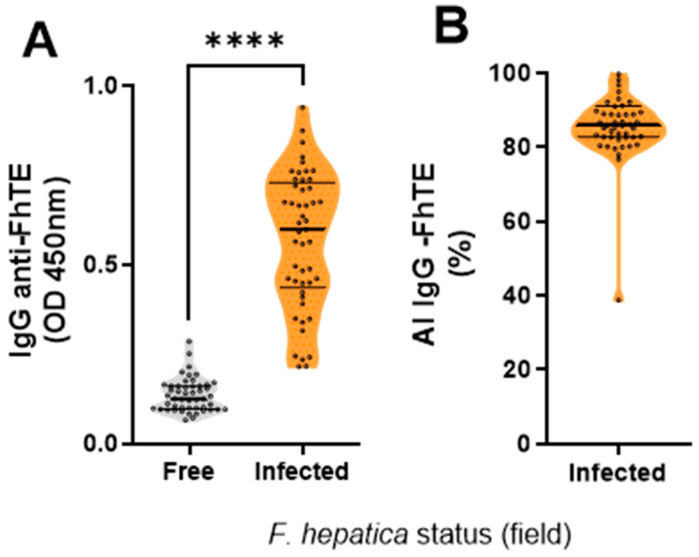
*F. hepatica*-specific antibodies in sera of infected (orange) and not-infected (grey) buffalo herds. (**A**) Parasite-specific IgG levels were detected by ELISA. (**B**) Avidity index of the antibodies. **** *p* < 0.0001. Mann–Whitney test.

IgG levels against *F. hepatica* proteins differed significantly between the infected and the fasciolosis-free farms (*p* < 0.0001; Figure 1A), confirming the accurate classification of the infected and non-infected groups. The avidity of *F. hepatica*-specific IgG was also assessed and was high and comparable among infected animals (Figure 1B), presumably as a result of repeated reinfection due to continuous exposure to the parasite.

### 3.2. Vaccine-Induced Humoral Immune Responses Against FMDV

**Figure 2:**
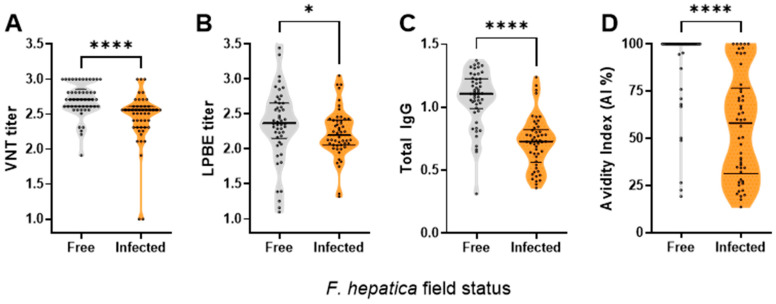
FMDV-A24/Cruzeiro specific antibodies in sera of *F. hepatica*-infected (orange) and -free (grey) buffalo herds. (**A**) Neutralising antibody titres. (**B**) Total antibody titres (LPBE). Antibodies specific to the FMDV A24/Cruzeiro strain were also detected by indirect ELISAs using 146S purified viral particles as capture antigen: (**C**) total IgG and (**D**) avidity index of IgG. Dots depict mean values for each individual animal. Group mean values and SD are indicated with horizontal lines. Asterisks indicate statistically significant differences between infected and not-infected animals calculated with the Mann–Whitney test: * *p* < 0.01, **** *p* < 0.0001.

Several parameters of the FMDV-specific humoral immune response were assessed using the A24/Cruzeiro vaccine strain study as a proxy for the overall humoral response to FMDV [6,22]. Across all measured parameters, antibody responses were lower in *F. hepatica*-infected buffaloes compared with non-infected animals. Mean log_10_ virus-neutralising antibody titres were significantly reduced in *F. hepatica*-infected buffaloes (2.4 ± 0.36; 95% CI: 2.30–2.50) relative to non-infected animals (2.7 ± 0.22; 95% CI: 2.64–2.76), corresponding to an approximately two-fold reduction in neutralising capacity (10^0.3^ ≈ 2) (Figure 2A). Although variability was higher in the infected group (CV 15% vs. 8%; Table 1), confidence intervals did not overlap.

In the non-infected group, 8% of the animals displayed LPBE titres below 1.9, corresponding to the EPP75% estimated for cattle (Figure 2B), with 8% showing low total IgG levels (Figure 2C) and only 6% exhibiting low avidity indexes (Figure 2D). In contrast, among the infected group, only 18% of the animals had LPBE titres greater than 1.9. Low avidity indexes of the specific IgG were found in 34% of the *F. hepatica*-infected animals; 4% displayed low VNT titres (Figure 2A), and 54% had total IgG levels below the protective threshold.

The mean avidity index in infected buffaloes was 56.2% (CV 49.7%; 95% CI: 48.5–63.9), whereas non-infected animals showed a mean avidity index of 90.0% (CV 23.6%; 95% CI: 84.6–96.4). The difference between groups corresponded to an approximate 38% reduction in IgG avidity in infected animals. Although variability was higher in the infected group (Table 1), confidence intervals did not overlap.

The mean values for all the FMDV-specific serological parameters were lower in the infected group compared to the non-infected group, with a higher coefficient of variation (Table 1). Only LPBE titres and total IgG levels against A24/Cruzeiro followed a normal distribution (*p* > 0.05; D’Agostino–Pearson test). Table 1 also depicts that the distribution of IgG avidity indexes, VNT titres, IgG levels, and total antibody titres (LPBE) in infected animals displayed a negative skewness index, suggesting a concentration of values toward the lower end of each parameter. In contrast, a symmetric distribution was observed in non-infected animals. Kurtosis indexes further indicated that the serological parameters in the infected group were more dispersed around the mean compared to the non-infected group.

## 4. Discussion

This study provides field-based evidence that natural fasciolosis is associated with alterations in the humoral immune response induced by foot-and-mouth disease virus (FMDV) vaccination in water buffaloes. Infected animals exhibited lower total antibody levels, antibody avidity, and virus-neutralising activity, suggesting that helminth-associated immunomodulation affects both the magnitude and quality of vaccine-induced humoral immunity. These findings are in line with our previous experimental observations in cattle [19] and extend them to water buffaloes, a species of epidemiological relevance for FMD [1,3,23] in which vaccine-induced immune responses under field conditions remain poorly characterised.

Assessment of vaccine-induced humoral immunity in water buffaloes commonly relies on immunoassays originally developed and validated in cattle [16]. However, increasing evidence suggests that antibody thresholds and qualitative parameters established for cattle may not be fully transferable to buffaloes [6,24]. In this context, the inclusion of virus neutralisation tests and antibody avidity assays [24,25] in the present study provided functionally relevant and complementary measures of vaccine-induced immunity maturation, allowing a more comprehensive evaluation of humoral response quality beyond total antibody titres alone.

The reductions observed in antibody avidity, neutralising activity, and total Ig and IgG levels in *F. hepatica*-infected animals indicate a coordinated effect on humoral immunity rather than isolated alterations in individual assays. This pattern is consistent with the well-documented immunomodulatory properties of helminth infections, which are commonly related to a regulatory-biased immune environments [9], potentially compromising the generation of effective vaccine-induced humoral responses [7,19] and have been shown to interfere with antigen presentation and CD4^+^ T-cell-dependent processes in ruminants infected with *Fasciola* spp. [26,27]. The concordant impact across quantitative and functional parameters supports the interpretation of a system-wide modulation of vaccine-induced humoral immunity.

Virus-neutralising titres remained above cattle-derived protective thresholds [16] in both infected and uninfected groups, likely reflecting the administration of three vaccine doses. This observation suggests that repeated vaccination may sustain population-level immunity under field conditions, even in the presence of immunomodulatory pressures, and highlight the value of assays detecting differences in immune quality.

Although it was not possible to evaluate primary immune responses in infected buffalo calves, as animals under one year of age were not infected with *F. hepatica*, this does not represent a major limitation in the Argentine context, where primary FMD vaccination is routinely administered early in life. Consequently, the study design accurately reflects the prevailing vaccination and exposure conditions encountered in endemic field settings.

Natural *F. hepatica* exposure is characterised by repeated or prolonged infection, as reflected by high-avidity anti-*F. hepatica* antibodies, in contrast to the decay typically observed after single experimental infections [9]. We have previously demonstrated that the observed impairment in immune responsiveness is linked to the systemic reactivation of the parasite, a phenomenon that may occur continuously under field conditions. In this context, infected buffaloes consistently showed lower neutralising titres, antibody avidity, Ig titres, and total IgG levels following FMD vaccination, despite field-associated biological heterogeneity, indicating concordant modulation of humoral immunity.

This study provides the first field-based evidence that natural *F. hepatica* infection is associated with altered FMD vaccine-induced humoral immunity in water buffaloes. Given the roaming nature of buffaloes and their proximity to regions neighbouring Argentina where FMD vaccination has stopped, these animals should be a primary target for surveillance. The observed differences in vaccine-induced humoral responses indicate that *F. hepatica* infection may influence the interpretation of post-vaccination serological monitoring in water buffaloes. Addressing these factors is essential to ensure adequate vaccine coverage and support FMD control efforts.

## 5. Conclusions

This study shows that natural fasciolosis is associated with changes in FMD vaccine-induced humoral responses in water buffaloes under field conditions. Accounting for this interaction may improve the interpretation of post-vaccination serological monitoring in buffalo populations.

## Figures and Tables

**Table 1:** FMDV antibody response in buffaloes infected or not infected with *F. hepatica*. Mean values (Mean) and coefficient of variation (CV) are indicated for each parameter tested. Skewness and kurtosis results are also depicted.

Serological Test (FMDV)		IgG Avidity Index		Neutralising Titre		IgG Level		LPBE Titre	
*F. hepatica* Status		Free	Infected	Free	Infected	Free	Infected	Free	Infected
Parameters	Mean	90.5	56.2	2.7	2.5	1.1	0.7	2.4	2.2
	CV	23.5	49.7	8.7	15.3	28.5	20.1	15.3	22.3
	Skewness Index	0.21	−2.4	0.8	−2.3	0.4	−1.2	0	−0.4
	Kurtosis Index	−1.3	4.7	1.5	7.9	0.2	2.0	0.7	1.2

## Data Availability

The data supporting the findings of this study will be made available by the authors on request.
